# Epidermal growth factor receptor regulates β-catenin location, stability, and transcriptional activity in oral cancer

**DOI:** 10.1186/1476-4598-9-64

**Published:** 2010-03-19

**Authors:** Chien-Hsing Lee, Hsing-Wen Hung, Pei-Hsin Hung, Yi-Shing Shieh

**Affiliations:** 1 Graduate Institute of Medical Sciences, National Defense Medical Center, Taipei, Taiwan; 2 Department of Internal Medicine, Tri-Service General Hospital, Taipei, Taiwan; 3 School of Dentistry, National Defense Medical Center, Taipei, Taiwan; 4 Department of Oral Diagnosis and Pathology, Tri-Service General Hospital, Taipei, Taiwan

## Abstract

**Background:**

Many cancerous cells accumulate β-catenin in the nucleus. We examined the role of epidermal growth factor receptor (EGFR) signaling in the accumulation of β-catenin in the nuclei of oral cancer cells.

**Results:**

We used two strains of cultured oral cancer cells, one with reduced EGFR expression (OECM1 cells) and one with elevated EGFR expression (SAS cells), and measured downstream effects, such as phosphorylation of β-catenin and GSK-3β, association of β-catenin with E-cadherin, and target gene regulation. We also studied the expression of EGFR, β-catenin, and cyclin D1 in 112 samples of oral cancer by immunostaining. Activation of EGFR signaling increased the amount of β-catenin in the nucleus and decreased the amount in the membranes. EGF treatment increased phosphorylation of β-catenin (tyrosine) and GSK-3β(Ser-(9), resulting in a loss of β-catenin association with E-cadherin. TOP-FLASH and FOP-FLASH reporter assays demonstrated that the EGFR signal regulates β-catenin transcriptional activity and mediates cyclin D1 expression. Chromatin immunoprecipitation experiments indicated that the EGFR signal affects chromatin architecture at the regulatory element of cyclin D1, and that the CBP, HDAC1, and Suv39h1 histone/chromatin remodeling complex is involved in this process. Immunostaining showed a significant association between EGFR expression and aberrant accumulation of β-catenin in oral cancer.

**Conclusions:**

EGFR signaling regulates β-catenin localization and stability, target gene expression, and tumor progression in oral cancer. Moreover, our data suggest that aberrant accumulation of β-catenin under EGFR activation is a malignancy marker of oral cancer.

## Background

The Wnt/β-catenin pathway plays important roles in morphogenesis, normal physiological functions, and tumor formation. At the molecular level, β-catenin is involved in two apparently independent processes, cell-cell adhesion and signal transduction [1]. In the absence of a mitotic signal, β-catenin is sequestered in a "destruction complex" which consists of the adenomatous polyposis coli (APC) gene product, casein kinase 1 (CK1), a serine threonine glycogen synthetase kinase (GSK-3β), and axin, an adapter protein [2]. This destruction complex is phosphorylated and degraded by the ubiquitin-proteasome system [2]. β-catenin also plays a role in the transcription activation pathway [3,4]. Following stimulation of mitosis signal, β-catenin accumulates in the cytoplasm, moves to the nucleus, and then binds to a member of the TCF/LEF-1 family of transcription factors that modulate expression of TCF/LEF-1 target genes [5-7]. Previously, we and others reported that aberrant expression of β-catenin was common in oral cancer and this change correlated with the malignancy index and patient prognosis [8,9]. However, the molecular mechanisms that lead to aberrant expression of β-catenin in oral cancer are unclear, and the mechanisms by which β-catenin promotes activation of target genes are also not well understood.

Certain mutations of APC or β-catenin increase β-catenin signaling, leading to overexpression of oncogenes and promotion of neoplastic growth [10-15]. However, for some cancers, β-catenin accumulates in the nucleus even though mutation of β-catenin or APC is rare. For example, in endometrial cancers, 12 of 20 cases (60%) exhibited β-catenin accumulation in the nucleus, but only two of these cases had mutations in the β-catenin gene [16]. In hepatocellular carcinomas, nearly 50% of cases exhibited nuclear accumulation of β-catenin, but APC mutation was very rare and only 16-26% of cases had mutations in β-catenin [10,17-19]. Similar findings have been reported for oral cancer [8]. Therefore, it is possible that mechanisms other than mutation are involved in the aberrant β-catenin expression observed in tumors.

Recent reports have suggested that receptor tyrosine kinases (RTKs) can regulate β-catenin function [20,21]. Epidermal growth factor receptor (EGFR) is a member of the receptor tyrosine kinase family, and overexpression of EGFR is associated with poor prognosis and progression of many human cancers, including oral cancer [22,23]. At the molecular level, stimulation of EGFR induces intrinsic tyrosine kinase activity and cellular signaling that results in cell growth and proliferation. EGFR stimulation is associated with perturbation of E-cadherin-mediated cell adhesion, development of fibroblast-like morphology, and increased cell motility in certain tumors [24-26]. Moreover, EGFR interacts with the β-catenin core region and induces tyrosine phosphorylation of catenins in several types of tumors [27,28]. This raises the possibility that EGFR signaling may play a role in the regulation of β-catenin. It is not yet known whether EGFR plays a role in the aberrant expression of β-catenin that is seen in oral cancer.

In the present paper, we describe the effect of EGFR signaling on the nuclear accumulation of β-catenin in oral cancer. This extends our previous research into the mechanisms that underlie aberrant accumulation of β-catenin.

## Methods

### Cell culture and reagents

All cell lines were maintained in DMEM or RPMI1640 media that were supplemented with 10% bovine serum and 1% gentamycin. Cells were maintained in a humidified atmosphere containing 5% CO_2 _at 37°C and the medium was changed three times per week. Cell lines were grown until they were 89-90% confluent. All cultures were negative for mycoplasma infection.

Recombinant human EGF was obtained from R&D Systems (Minneapolis, MN, USA), EGFR inhibitor (AG1478) from A.G. Scientific (San Diego, CA, USA), lithium chloride from Acros Organics Co. (Geel, Belgium), Erbitux from Merck (Darmstadt, Germany), mouse anti-E-cadherin and mouse anti-β-catenin from BD Transduction Lab (Lexington, KY, USA), phospho-GSK-3β (Ser-9), phorpho-β-catenin (Ser33/37/Thr41), EGFR antibody, and phosphor-tyrosine antibody from Cell Signaling Technologies (Beverly, MA, USA), anti-HDAC1, anti-cyclin D1, goat anti-rabbit IgG-HRP, donkey anti-goat IgG-HRP, and protein A/G Plus-Agarose immunoprecipitation reagent from Santa Cruz Biotechnology (CA, USA), anti-Suv39h1 (05-615), anti-acetyl histone H4 (06-88-66), anti-trimethyl-histone H3K9 (07-442), and anti-trimethyl-histone H3K4 (07-473) from Upstate Chemicon (Temecula, CA, USA), rabbit anti-mouse IgG conjugated to HRP antibody from Novus Biologicals (Littleton, CO, USA), anti-human EGFR and anti-CBP, anti-Lamin B1, and anti-alpha-tubulin from Abcam (Cambridge, UK).

### Patients and tissue specimens

All specimens were obtained from the archives of Tri-Service General Hospital (Taipei, Taiwan) and included 112 samples of oral squamous cell carcinoma (HNSCC). The study design was approved by the Internal Review Board of Tri-Service General Hospital (TSGHIRB 095-05-116). More detailed information about the specimens was provided previously [29]. A series of 5-μm sections were cut from each tissue block. A 5 μm flanking section was stained with hematoxylin and eosin (H&E) for pathological evaluation and to identify the cancerous and normal regions. Serial sections were used for immunohistochemistry (IHC).

### Cell fractionation and Western blotting

Cellular fractionation was performed as described previously [30]. Briefly, cells were washed twice with ice-cold phosphate-buffered saline, harvested by scraping with a rubber policeman, and lysed in a buffer (20 mM HEPES, pH 7.0, 10 mM KCl, 2 mM MgCl_2_, 0.5% Nonidet P-40, 1 mM Na_3_VO_4_, 10 mM NaF, 1 mM phenylmethanesulfonyl fluoride, 2 μg/mL aprotinin). After incubation on ice for 10 min, the cells were homogenized by 20 strokes in a tightly fitting Dounce homogenizer. The homogenate was centrifuged at 1,500 × *g *for 5 min to sediment the nuclei. The supernatant was then centrifuged at 16,000 × *g *for 20 min, with the resulting supernatant considered the non-nuclear fraction. The nuclear pellet was washed three times with lysis buffer to remove contamination from cytoplasmic membranes. To extract nuclear proteins, isolated nuclei were resuspended in NETN buffer (150 mM NaCl, 1 mM EDTA, 20 mM Tris-Cl, pH 8.0, 0.5% Nonidet P-40, 1 mM Na_3_VO_4_, 10 mM NaF, 1 mM phenylmethanesulfonyl fluoride, and 2 μg/mL aprotinin), and the mixture was sonicated briefly to facilitate nuclear lysis. Nuclear lysates were collected after centrifugation (16,000 × *g *for 20 min at 4°C). Samples of each lysate were subjected to electrophoresis on an 8% SDS-polyacrylamide gel. Then, proteins were transferred to nitrocellulose membranes, immunoblotted with antibodies, and detected by electrochemiluminescence.

### Preparation of cell lysates and immunoprecipitation

Cell monolayers were rinsed with 1× Tris-based saline (TBS) and then scraped into 1 mL of TBS. After a brief centrifugation, cells were solubilized in 1 mL of cell lysis buffer (150 mM Tris-HCl, pH 7.4, 150 mM NaCl, 1 mM EDTA, 1% TRITON^® ^X-100 plus 1:100 protease inhibitor cocktail, P8340 from Sigma, and 1:100 phosphatase inhibitor cocktails, P5726 from Sigma). Before immunoprecipitation (IP), all samples were centrifuged at 12,000 × *g *for 30 minutes to remove insoluble cellular debris. For IP studies, lysates were pre-cleared for 1 h by use of protein A/G PLUS-agarose (sc-2003, Santa Cruz Biotechnology, CA, USA), incubated with antibodies at 4°C, and then treated with protein A/G PLUS-agarose for an additional 1 h. Immunoprecipitates were then washed 4 times with 1 mL TBS. After heating at 95°C for 10 minutes, proteins were resolved on SDS-PAGE, transferred to PVDF membranes for Western blot analysis, and immunoblotted with antibodies.

### Luciferase reporter assays

SAS and OECM1 oral cancer cells were plated in 24-well dishes and incubated overnight at 37°C. The following day, cells were transfected with 1 μg of β-catenin-LEF/TCF-sensitive (TOP) or β-catenin-LEF/TCF-insensitive (FOP) reporter vector using Lipofectamine 2000 (Invitrogen, Carlsbad, CA, USA) according to the manufacturer's instructions. On the following day, cells were washed with serum-free medium and treated with EGF (OECM1 cells; 100 ng/μL) or AG1478 (SAS cell; 20 μM). Reporter assays were performed using the luciferase reporter system (Promega, Madison, WI, USA).

### Chromatin immunoprecipitation assay

The chromatin immunoprecipitation assay (ChIP) was performed using a kit from Upstate (Lake Placid, NY, USA) according to the manufacturer's instructions. Briefly, following treatment, cells were washed with PBS, cross-linked with 1% formaldehyde for 10 min, rinsed with ice-cold PBS, collected into PBS containing protease inhibitors, and then resuspended in lysis buffer (1% SDS, 10 mM EDTA, 50 mM Tris at pH 8.1 with 1% protease inhibitor cocktails). Cells were sonicated to produce 200-1000 bp of DNA fragments, followed by centrifugation to remove insoluble material. Samples were precleared for 1 h at 4°C with 60 μL of a 50% slurry of protein G agarose and salmon sperm. DNA immunoprecipitation was performed with indicated antibodies. Then, cross-links were reversed, and the bound DNA was purified by phenol:chloroform extraction. RT-PCR was performed using primers specific for human cyclin D1 promoter (5'-CCGACTGGTCAAGGTAGGAA-3' and 5'-CCAAGGGGGTAACCCTAAAA-3'). PCR reactions were run with PCR Master Mix (Promega), which consisted of 30 cycles of: 94°C × 30 s, 55°C × 30 s, and 72°C × 1 min, followed by 5 min at 72°C. PCR products were analyzed by 1.5% agarose gel electrophoresis, visualized with ethidium bromide, and then photographed. Images were saved as TIFF files and then analyzed with ImageJ http://rsb.info.nih.gov/ij/. Signal intensities of the PCR data obtained from ChIP assays or from whole-cell lysates (Input DNA) were quantified from TIFF images by use of ImageJ, and then compared to the signal obtained for input control. Each ChIP experiment was repeated at least three times, and representative results are shown. Means and standard deviations (SDs) were calculated from the signal intensities.

### Immunohistochemistry

Specimens that were embedded in paraffin blocks were cut into 5-μm sections. These were routinely stained with H&E for histological diagnosis, and additional sequential sections were selected for immunohistochemical studies. Immunodetection was performed with a standard DAKO EnVision stain system (Dako Corp, Carpinteria, CA, USA). Sections were dewaxed and subjected to antigen heat retrieval. Endogenous peroxidase activity and nonspecific binding were blocked by incubation with 3% hydrogen peroxide and nonimmune serum, respectively. Slides were then incubated sequentially with primary antibodies (16 h at 4°C) and DAKO labeled polymer secondary antibody (1 h at room temperature, then peroxidase-labeled polymer (30 min at room temperature). Diaminobenzidine hydrochloride (DAB) was used to visualize peroxidase activity. Then, sections were counterstained with hematoxylin and a cover slip was added prior to visualization.

### Assessment of immunoreactivity

Using a semi-quantitative scale described previously [8], the staining results of EGFR and cyclin D1 were classified as "high" or "low" staining. Briefly, in the clyclin D1 staining, tumors were evaluated as high if more than 10% of cells displayed nuclear staining and as low if otherwise. For the EGFR staining, scores representing the percentage of stained cancer cells were as follows: 0, no stained cells; 1, 1%-30%; 2, 31%-50%; and 3, >50%. Intensity was graded from 0 (no staining) to 3 (strong) in comparison with normal epithelium. Tumors were defined as high EGFR expression if the final score was 5 or 6 and as low if otherwise. The staining results for β-catenin were classified as membranous or cytoplasmic/nuclear, as in a previous report [8]. Briefly, tumors were regarded as cytoplasmic/nuclear stain if unequivocal cytoplasmic and/or nuclear staining was present in at least one area of the tumor, and membranous stain if β-catenin was localized solely in the membrane. Immunostaining results were evaluated by two investigators (YSS and LCC) who had no prior knowledge of the histopathologic features of the tumor or the clinical status of the patient from whom the cell lines were obtained.

### Statistical analysis

A χ^2 ^test was used to assess the relationship of the results of the immunohistochemical determination of EGFR, β-catenin, and cyclin D1 expression and the clinical features of patients. *P *values less than 0.05 were considered statistically significant.

## Results

### APC and β-catenin mutation in oral cancer cell lines

In many cancers, activation of the Wnt/β-catenin pathway is associated with mutations of APC and β-catenin, with exon 15 of APC and exon 3 of β-catenin the most common mutation sites [10,31]. Our initial examination of five oral cancer cell lines (SAS, SCC25, YD8, YD38, and OECM1) found no evidence of mutations of APC or β-catenin (data not shown).

### EGFR signal-mediated subcellular localization of β-catenin

**Figure 1:**
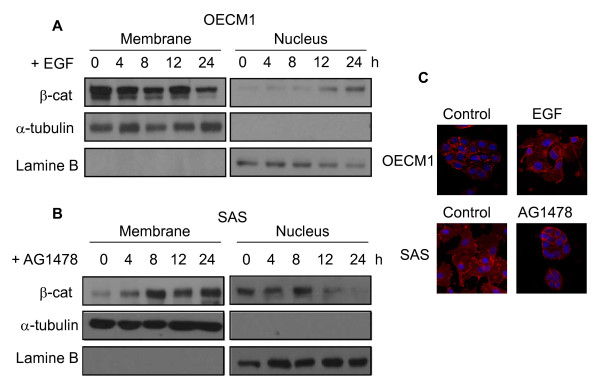
**Effect of EGFR on the subcellular distribution of β-catenin in oral cancer cell lines**. Time-dependent effects of EGF or AG1478 on the subcellular distribution of β-catenin in OECM1 (A) and SAS (B) cells. Cancer cells were treated with EGF (100 ng/mL) or AG1478 (20 μM/mL) for the indicated time, and β-catenin was assayed by Western blotting. (C) Immunocytochemical staining of β-catenin in oral cancer cells. EGF treatment of OECM1 cells induced scattering of cancer cells, breakup of cell-cell junctions, and decreased level of membranous β-catenin (upper panels). AG1478 treatment of SAS cells led to close cell-to-cell contact and abundant membranous β-catenin (lower panels). Immunofluorescence for β-catenin (Rhodamine, Red) and the nucleus (Dapi) in cultured cells was performed 24 h following treatment. Cells were permeabilized with 100% methanol, blocked with 1% BSA, and incubated with the antibody for 30 min. A rhodamine conjugated antibody was used as the secondary antibody.

To investigate the effect of the EGFR signal on β-catenin redistribution, we used two lines of oral cancer cells, SAS and OECM1. SAS cells have elevated levels of EGFR, and were used for loss-of-function assays; OECM1 cells have reduced levels of EGFR and were used for gain-of-function assay. When OECM1 cells were treated with EGF, which activates EGFR, the amount of nuclear β-catenin increased over time (Figure 1A). After one day, cells became elongated and spindle-shaped (resembling mesenchymal cells) and had greatly reduced cell-cell contacts (Figure 1C). When SAS cells were treated by AG1478, which inhibits the EGFR signal, there was a decrease of nuclear β-catenin, and an increase in membranous β-catenin (Figure 1B), and cells had the typical epithelial phenotype, with close cell-to-cell contacts (Figure 1C).

### EGFR signal- induced phosphorylation of β-catenin and GSK-3β

**Figure 2:**
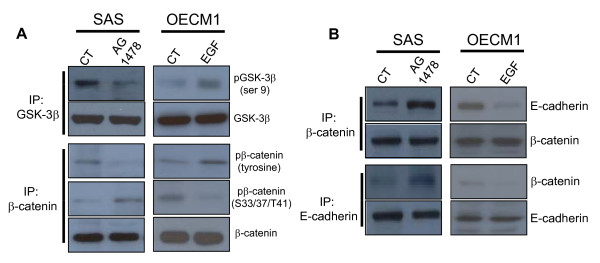
**Effects of EGFR signal on the phosphorylation and function of β-catenin and GSK-3β**. (A) AG1478 treatment of SAS cells decreased phosphorylation of GSK-3β (Ser-9) and β-catenin (Tyr). EGF treatment of OECM1 cells increased phosphorylation of GSK-3β (Ser-9) and β-catenin (Tyr), as determined by immunoprecipitation of GSK-3β or β-catenin. (B) In SAS cells, association of E-cadherin and β-cadherin increased following treatment with AG1478; in OECM1 cells, association of E-cadherin and β-catenin decreased following treatment with EGF as determined by immunoprecipitation of E-cadherin or β-catenin.

To determine whether the phosphorylation of β-catenin or GSK-3β is associated with the nuclear translocation of β-catenin, we treated cells with AG1478 or EGF and examined the phosphorylation status of β-catenin and GSK-3β. Treatment of SAS cells with AG1478 markedly suppressed the phosphorylation of GSK-3β (Ser-9) and β-catenin (tyrosine) (Figure 2A); treatment of OECM1 cells with EGF increased the phosphorylation of GSK-3β(Ser-9) and β-catenin (tyrosine) (Figure 2A).

GSK-3β phosphorylates cytosolic β-catenin at Ser-33, Ser-37, and Thr-41 prior to β-catenin degradation, but phosphorylation of GSK-3β at Ser-9 inhibits its kinase activity. Thus we measured the phosphorylation of β-catenin at these three residues by use of phosphorylation-specific antibodies. As shown in Figure 2A, AG1478 increased the phosphorylation of β-catenin at all three sites in SAS cells, but treatment with EGF led to decreased phosphorylation of all three sites in OECM1 cells. Then we examined the effect of EGFR on the formation of complexes of E-cadherin and β-catenin. The results indicate that inhibition of EGFR by AG1478 increased the amount of β-catenin that was associated with E-cadherin in SAS cells (Figure 2B), and that EGF decreased the amount of β-catenin associated with E-cadherin in OECM1 cells (Figure 2B). We observed similar results when performing immunoprecipitation for E-cadherin (Figure 2B).

### Effect of EGFR signal on transcription activity of β-catenin

**Figure 3:**
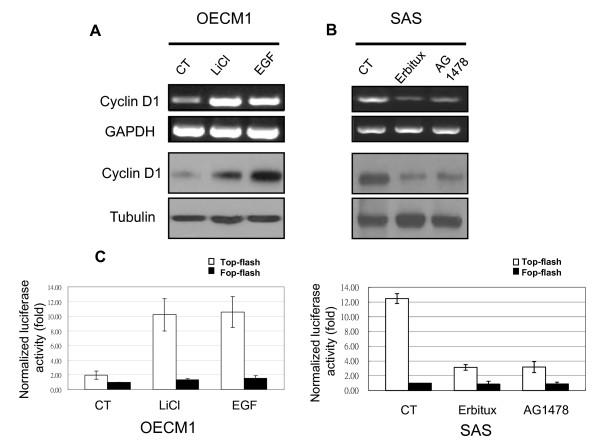
**EGFR signaling regulates cyclin D1 expression and β-catenin/TCF transcription activity**. (A) Cyclin D1 expression increased when OECM1 cells were treated with EGF or LiCl (a GSK-3β inhibitor). (B) SAS cells treated with AG1478 or Erbitux had suppressed cyclin D1 expression. RNA and protein were isolated 24 h after treatment and subjected to RT-PCR and Western blotting. (C, D) TOP-FLASH or FOP-FLASH were transfected into OECM1 (C) or SAS (D) cells which were then treated with EGF, LiCl, AG1478, or Erbitux. Luciferase activity was determined after 24 h. Relative changes in expression are shown compared with the levels of FOP-FLASH in untreated (control) cells. Data represent means ± SDs of three independent experiments. Column, mean fold change; bar, SD.

Activation of the EGFR signal leads to nuclear translocation of β-catenin. Thus, we determined whether β-catenin-mediated promotion of transcription in cancer cells also depended on EGFR activity. Nuclear-localized β-catenin interacts with transcription factors of the TCF family, leading to increased expression of genes such as cyclin D1. Therefore, we tested RNA and protein levels of cyclin D1 using RT-PCR and Western blotting. EGF markedly stimulated cyclin D1 expression in OECM1 cells, and LiCl (a GSK-3β inhibitor) had a similar effect (Figure 3A). In contrast, AG1478 markedly decreased cyclin D1 expression in SAS cells, and Erbitux (an EGFR monoclonal antibody) had a similar effect (Figure 3B).

Next, we examined the effect of the EGFR signal on TCF transcriptional activity by transfected with the TCF luciferase reporter (TOP-FLASH) or a control vector (FOP-FLASH) in cells. Activated EGFR signal increased TCF transcriptional activity in OECM1 cells (Figure 3C), and inhibition of the EGFR signal suppressed transcription activity in SAS cells (Figure 3D).

### Histone modification and chromatin remodeling in the regulation of cyclin D1 expression

**Figure 4:**
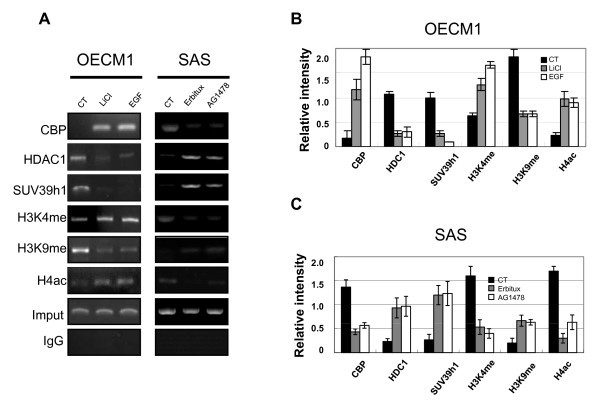
**ChIP assay in the cyclin D1 regulation element**. (A) OECM1 cells treated with EGF or LiCl, and SAS cells treated with AG1478 or Erbitux. (B, C) Quantification of proteins is described in "Materials and Methods" (B, OECM1 cells; C, SAS cells). (CT, control; H3K4me, H3K4 methylation; H3K9me, H3K9 methylation; H4ac, H4 acetylation).

Transcriptional activation is preceded by the formation of an activation complex with ATP-dependent chromatin remodeling enzymes and histone acetyltransferase in the promoter regions. Thus, we performed ChIP assays with primers that encompassed the cyclin D1 promoter region to test for site-specific histone modification and chromatin remodeling in the mediation of EGFR-regulated cyclin D1 expression. In OECM1 cells treated with EGF or LiCl, there was significantly increased association of the cyclin D1 promoter with CBP (a transcriptional co-activator) and a decrease in HDAC1 (a histone deacetylase) and Suv39h1 (a histone methyltransferase) (Figure 4A). In addition, analysis of histones in this area indicated an increase of methylated histone H3K4 and acetylated histone H4, and a decrease of methylated histone H3K9 (Figure 4A). Treatment of SAS cells with AG1478 or Erbitux significantly decreased CBP, methylated histone H3K4, acetylated histone H4, and increased HDAC1, Suv39h1, and methylated histone H3K9 association with the cyclin D1 promoter.

### Association of EGFR, β-catenin, and cyclin D1 immunostaining in oral cancer

**Figure 5:**
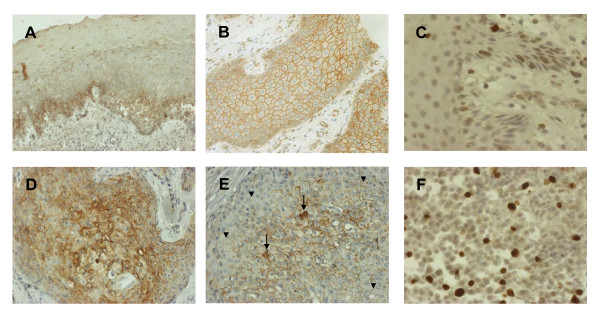
**Immunohistochemical staining of EGFR, β-catenin, and cyclin D1 in oral cancer and adjacent normal tissues**. EGFR-positive cells located in the basal and parabasal layer of a normal epithelium (A). β-catenin exhibited homogeneous expression in the membrane (B), and cyclin D1 expression was weak/undectectable (C). In tumor tissues, there was an increase of intensity and percentage of EGFR staining (D) and a loss of membranous stain (arrow head), increased cytoplasmic and nuclear accumulation (arrow) of β-catenin (E), and increased positive-staining cells of cyclin D1 (F). (Original magnifications; A, D: ×-20; B, E: ×-100; C, F: ×-400)

**Figure 6:**
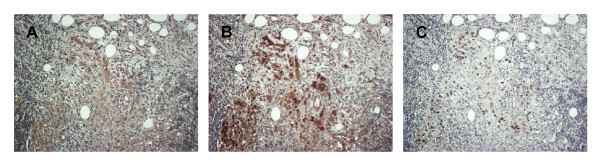
**Immunohistochemical staining of EGFR, β-catenin, and cyclin D1 in oral cancer**. Representative serial sections showed the area in tumor with high EGFR immunoreactivity (A) that was accompanied by cytoplasmic and nuclear β-catenin staining (B) and high cyclin D1 immunostaining (C). (Original magnifications; A, B, C: ×-20).

To investigate the clinical significance of our results with cultured cells, we performed immunohistochemical analysis of 112 samples of oral cancer and adjacent normal epithelium. In normal epithelial cells, there was weak expression of EGFR in basal and parabasal layers, homogeneous membranous staining of β-catenin, and rare or undetectable presence of cyclin D1 (Figures 5A-C). In tumor cells, there was elevated EGFR immunoreactivity in most samples (Figure 5D), decreased membranous staining and increased cytoplasmic/nuclear staining of β-catenin (Figure 5E), and positive staining for cyclin D1 (Figure 5F). Notably, in some serial sections of tumor cells, there was high EGFR immunoreactivity that was accompanied by cytoplasmic and nuclear β-catenin staining and high cyclin D1 immunostaining (Figure 6).

We observed reduced EGFR immunostaining in 66 samples (59%), and elevated expression of EGFR in 46 samples (41%). A blinded observer scored 86 samples (77%) as membranous β-catenin staining and 26 cases (23%) as cytoplasmic/nuclear β-catenin staining. The observer scored 59 samples (53%) as having low cyclin D1 staining and 53 cases (47%) with high cyclin D1 staining.

We examined the association of these results with various clinicopathologic features of the patients (Table 1). We found significant correlation of EGFR expression and tumor stage (P = 0.042), β-catenin and tumor size (P = 0.025) and stage (P = 0.031), and of EGFR expression and β-catenin cytoplasmic/nuclear expression and cyclin-D1 immunoactivity (P < 0.0001 for both) (Table 2). However, there was no significant correlation between β-catenin and cyclin D1 expression.

**Table 1:** Association of clinical features of patients and immunohistochemical expression of EGFR, β-catenin, and cyclin D1.

	EGFR expression		β-catenin expression		Cyclin D1 expression	
			
	Low	High	Mem	C/N	Low	High
Clinicopathologic features	(n = 66)	(n = 46)	(n = 86)	(n = 26)	(n = 59)	(n = 53)
Gender						
Male (n = 93)	56	37	70	23	55	38
Female (n = 19)	10	9	16	3	9	10
Size						
≤4.0 cm (n = 66)	43	23	55	11*	37	29
> 4.0 cm (n = 46)	23	23	31	15	27	19
LN involvement						
No (n = 64)	33	31	17	23	38	26
Yes (n = 48)	22	26	18	14	21	27
Differentiation						
Well (n = 41)	28	13	32	9	23	18
Moderate (n = 43)	22	21	32	11	25	18
Poor (n = 28)	16	12	22	6	16	12
Staging						
Early (n = 50)	35	15**	43	7***	30	20
Advanced (n = 62)	31	31	43	19	34	28

LN, lymph node; Mem, membranous; C/N, cytoplasmic/nuclear. *, p = 0.042; **, p = 0.025; ***, p = 0.031

**Table 2:** Relationships among EGFR, β-catenin, and cyclin D1 expression

	β-catenin expression			Cyclin D1 expression		
				
	Mem	C/N	p value	Low	High	p value
EGFR						
Low (n = 66)	63	3	<**0.0001**	49	17	<**0.0001**
High (n = 46)	23	23		15	31	
β-catenin						
Mem				53	33	0.065
C/N				11	15	
Total				64	48	

Mem, membranous; C/N, cytoplasmic/nuclear.

## Discussion

Dysregulation of the Wnt/β-catenin signaling pathway has been linked to various human cancers, and this dysregulation is often associated with mutations in the β-catenin destruction complex components or in β-catenin itself [26,32]. However, β-catenin signaling is elevated in oral cancer cells even though mutations of APC and β-catenin are rare. This suggests that alternative mechanisms may contribute to β-catenin dysregulation. The present study demonstrated that the EGFR signal participates in the dysregulation of β-catenin in oral cancer. First, we found that the EGFR signal stabilized β-catenin and enhanced β-catenin nuclear accumulation by phosphorylated regulation. Moreover, we also showed that histone markers of open or repressed chromatin control the expression of cyclin D1, a β-catenin target gene. Finally, our study of oral cancer patients suggests that β-catenin-mediated cross-talk between EGFR and Wnt signaling may underlie the effect of EGFR during tumor development.

Numerous cell signals can impact β-catenin function. It was recently demonstrated that numerous oncogenic tyrosine kinases promote accumulation of β-catenin in the nuclei of different types of cancer [33-36]. EGFR is the most commonly overexpressed receptor tyrosine kinase in oral cancer [22]. The present study showed that an activated EGFR signal decreased membrane-bound β-catenin, increased nuclear accumulation of β-catenin, and induced mesenchymal cell morphology. This result was consistent with previous reports that the EGFR signal is associated with perturbation of E-cadherin-mediated cell adhesion, acquisition of fibroblast-like cell morphology, and increases in cell motility that are presumably related to tumor invasion and metastasis [37,38]. β-catenin plays a critical structural role in cadherin-based cell-cell adhesion and is also an essential coactivator of Wnt-mediated gene expression. The extent to which β-catenin participates in these two functions is controlled by the availability of β-catenin binding partners, and there is increasing evidence that these binding interactions are regulated by phosphorylation. For example, binding of β-catenin to E-cadherin and to α-catenin was substantially reduced when tyrosine in β-catenin was phosphorylated by EGFR [27,39]. Moreover full activation of GSK-3β generally requires phosphorylation of Tyr-216, whereas phosphorylation of Ser-9 inhibits GSK-3β activity. Therefore, our results suggest that the EGFR signal enhances accumulation of β-catenin in the nuclei of oral cancer cells directly, by phosphorylation of β-catenin, and indirectly, by stabilization of β-catenin through phorsphorylation and inhibition of GSK-3β.

The identification of many nuclear partners of β-catenin indicates that this protein functions as a transcription regulator by covalent modification of chromatin [40,41]. Many of these nuclear partners regulate chromatin structure by histone modification and chromatin remodeling. In the present study, the results of our ChIP assay demonstrated that an activated EGFR signal greatly increased the amount of CBP/P300 coactivator and reduced the amount of HDAC1 and Suv39h1 in the regulatory element of cyclin D1. A previous study showed that the central repeats of β-catenin (span R3-R10) is the region that interacts with TCF [42]. In the absence of a nuclear β-catenin, TCFs recruit Groucho (TLF1 in mammals), a long-range chromatin repressor that functions with histone deacetylases (HDACs) to compress local chromatin and inhibit transcription [43,44]. Upon stimulation, β-catenin enters the nucleus and competes with Groucho for TCF binding, thus replacing the repressor with an activation scaffold [45]. Our results showed that the extent of H3K4 methylation (H3K4me3) increased significantly following activation of cyclin D1 transcription by β-catenin in EGFR-activated cells, and that it gradually declined when the gene was inactivated in EGFR-inhibited cells. H3K4me3 is more common in active genes, and is believed to promote gene expression via recognition by transcription-activating effector molecules [46]. A recent study showed that H3K4me3 also regulates another β-catenin target gene, c-myc [47]. To the best of our knowledge, this is the first report to demonstrate β-catenin regulated cyclin D1 via histone modification/chromatin remodeling. Taken together, our results suggest that the EGFR signal promotes nuclear accumulation of β-catenin, which ultimately forms β-catenin-TCF complexes with histone-acetylating activity, and that these displace the repressor complexes. These β-catenin complexes remodel the chromatin structure of target gene promoters so that they are more accessible to the basal transcription machinery, thus enhancing transactivation of genes that leads to cellular responses.

The results of our experiments with cancer tissues corroborated the results of our experiments with cultured cells. In cancer tissues, EGFR expression correlated with the presence of nuclear β-catenin, and nuclear β-catenin correlated with the tumor malignancy index. This implicates the EGFR signal in mediating entry of β-catenin into the nucleus and progression of oral cancer. Our results are consistent with other studies which reported that nuclear β-catenin was present in 19-23% of oral cancer cells and associated with proliferation, invasiveness, and poor outcome of oral cancer [8,48]. In contrast, Gasparoni et al. reported that nuclear β-catenin was rare in oral cancer and found no clear association between intranuclear β-catenin and histopathological and malignancy indexes in vivo [49]. The discrepancies between these studies could be explained by their use of different antibodies and methodologies. Although we did not find a close association between expression of nuclear β-catenin and cyclin D1, we did observe an association of nuclear β-catenin with the amount of cyclin D1 expression in some samples. This may be because multiple mechanisms regulate cyclin D1 expression in oral cancer cells [50,51]. For example, it is known that cyclin D1 amplification participates in overexpression of this gene in oral cancer [52,53]. Thus, in oral cancer, overexpression of cyclin D1 is more common than nuclear β-catenin expression (42% vs. 23%) [8]. Taken together, EGFR activation is an alternative mechanism that induces β-catenin translocation to the nucleus of certain oral cancer cells. We suggest that measurement of the activation of this pathway may be a useful marker for measuring the progression of oral cancer.

## Conclusions

**Figure 7:**
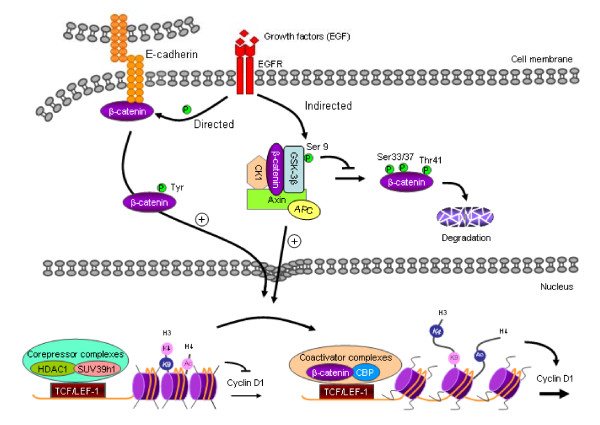
**Proposed model of a EGFR/β-catenin/cyclin D1 signaling pathway in oral cancer**. EGFR induces phosphorylation of β-catenin (Tyr) and GSK-3β (Ser-9). Phosphorylation of β-catenin (Tyr) leads to its dissociation from membranes. Phosphorylation of GSK-3β (Ser-9) inhibits its kinase activity so that β-catenin is not degraded, but is translocated to the nucleus. In the absence of an EGFR signal, β-catenin target genes are occupied by a repression complex (e.g., HDAC and Suv39h1), and histones in this area are in a compressed status, marked by high level of H3K9 methylation. Following EGFR signal activation, nuclear-translocated β-catenin combines with TCF/LEF transcription factors to recruit coactivators (e.g., CBP, Brg, etc.) to the regulatory element. These replace the repression complexes, leading to decompression of chromatin and high level of histone acetylation and H3K4 methylation.

In summary (Figure 7), our study demonstrated that, in addition to mutation of APC and β-catenin, oncogenic changes downstream of EGFR play important roles in regulating the nuclear translocation of β-catenin, a process that remodels histone/chromatin binding regions in target genes, and ultimately leads to the progression of oral cancer.

## List of abbreviations

The abbreviations used are: APC: Adenomatosis polyposis coli; ATP: Adenosine-5'-triphosphate; CBP: CREB binding protein; ChIP: Chromatin immunoprecipitation; DMEM: Eagle's minimal essential medium; EGF: Epidermal growth factor; EGFR: Epidermal growth factor receptor; GSK-3β: Glycogen synthase kinase 3 beta;HDAC1: Histone deacetylase 1; H3K4: Histone 3 lysines 4; H3K9: Histone 3 lysines 9; IHC: Immunohistochemistry; LEF-1: Lymphoid enhancer factor-1; LiCl: Lithium chloride; RTKs: Receptor tyrosine kinases; RT-PCR: Reverse transcription polymerase chain reaction; Ser: Serine; Suv39h1: Suppressor of variegation 3-9 homolog 1; TCF: T-cell factor; Thr: Threonine; Tyr: Tyrosine.

## Competing interests

The authors declare that they have no competing interests.

## Authors' contributions

CHL performed the experiments and prepared the draft version of the manuscript. HWH and PHH participated part of the experiments and data analysis. YSS designed the experiments, supervised the project, and prepared the manuscript. All authors have read and approved the final version of the manuscript.
